# Supplementary material for: Effects of a BMI1008 mixture on postoperative pain in a rat model of incisional pain
Source: PLoS One. 2021 Sep 27;16(9):e0257267. doi: 10.1371/journal.pone.0257267 (PMC8476004; doi:10.1371/journal.pone.0257267)
Supplement: Attachment — Submitted filename: Review.docx [file pone.0257267.s002.docx]

This is an experimental study to confirm the control of incisional pain according to the dose of BMI1008 and BMI-L. However, authors need to correct the manuscript overall.

MP4, L84-85 . Please add a reference.

MP18, L390 "The additivity of lidocaine and BMI-L appeared more likely than

antagonism when these drugs were co-administered." Can this dose-response relationship be explained as antagonism? I cannot understand. Please explain more detail.

The discussion section is not organized in general. Rearrange all paragraphs. For example, you need not to elaborate on the( MP18, L396-399) incisional pain model. Rather, it would be better to find the literature on the dose-response relationship of drugs and add them. Or please add more details such as analgesic effect when used in actual anesthesia clinical practice

MP19 L410 It has been reported that vasoactive agents and alpha2-adrenergic agents increase the quality and duration of local anesthetics. For example, which drugs do you mean? Are there any drugs other than epinephrine that have such an effect?

MP21 464 Is it correct to use the word "hyperalgesia"? Do you mean "mechanical stimulation"?

It is very important to write in the correct terminology.

I have a question, are BMI1008 and BMI-L drugs that the authors prepared and used in the study? Is it a drug marketed under a brand name?
