# Supplementary material for: Effects of a BMI1008 mixture on postoperative pain in a rat model of incisional pain
Source: PLoS One. 2021 Sep 27;16(9):e0257267. doi: 10.1371/journal.pone.0257267 (PMC8476004; doi:10.1371/journal.pone.0257267)
Supplement: Attachment — Submitted filename: Response to Reviewers.docx [file pone.0257267.s003.docx]

Dear Editor, Plose one

Re: [PONE-D-21-13470] - [EMID:9c926866ae8b5e17] entitled “**Effects of a BMI1008 Mixture on Postoperative Pain in a Rat Model of Incisional Pain**”.

I sincerely thank editor and reviewers for taking precious time to review our paper. I earnestly tried to response for your letter. I want to express my heartfelt gratitude for your comments once more.

I have made some corrections in the manuscript after going over your comments. I highlighted the modification made to the original document by using red colored text. The changes are summarized below:

**Editor’s comment**

1. Please ensure that your manuscript meets PLOS ONE's style requirements, including those for file naming. The PLOS ONE style templates can be found at

https://journals.plos.org/plosone/s/file?id=wjVg/PLOSOne_formatting_sample_main_body.pdf and

[https://journals.plos.org/plosone/s/file?id=ba62/PLOSOne_formatting_sample_title_authors_affiliations.pdf](about:blank)

**Our response**: Thank you for editor’s comment. According to editor’s comment, we ensure that our manuscript meets PLOS ONE's style requirements.

2. 'To comply with PLOS ONE submissions requirements, please provide methods of sacrifice in the Methods section of your manuscript.

**Our response**: Thank you for editor’s comment. According to editor’s comment, we revised the manuscript.

On final day of experiment, rats were sacrificed in a carbon dioxide chamber (CO_2_) two to four hours before dark-phase onset.

**(Materials and Methods section; from line 122 to line 123 on page 8)**

3.  Thank you for stating the following in the Financial Disclosure section:

"HK:
BMI Korea supported BMI 1008 materials and research grants for this research.
No role in the study design, data collection and analysis, decision to publish, or
preparation of the manuscript.
GJC:
This research was supported by the Basic Science Research Program through the
National Research Foundation of Korea (NRF) funded by the Ministry of Education,
Science and Technology [Grant No. NRF-2020R1C1C1011263].
No role in the study design, data collection and analysis, decision to publish, or
preparation of the manuscript"

We note that you received funding from a commercial source: "BMI Korea"

Please provide an amended Competing Interests Statement that explicitly states this commercial funder, along with any other [relevant declarations](about:blank) relating to employment, consultancy, patents, products in development, marketed products, etc.

Within this Competing Interests Statement, please confirm that this does not alter your adherence to all PLOS ONE policies on sharing data and materials by including the following statement: "This does not alter our adherence to PLOS ONE policies on sharing data and materials.” (as detailed online in our guide for authors [http://journals.plos.org/plosone/s/competing-interests](about:blank)).  If there are restrictions on sharing of data and/or materials, please state these. Please note that we cannot proceed with consideration of your article until this information has been declared.

Please include your amended Competing Interests Statement within your cover letter. We will change the online submission form on your behalf.

Please know it is PLOS ONE policy for corresponding authors to declare, on behalf of all authors, all potential competing interests for the purposes of transparency. PLOS defines a competing interest as anything that interferes with, or could reasonably be perceived as interfering with, the full and objective presentation, peer review, editorial decision-making, or publication of research or non-research articles submitted to one of the journals. Competing interests can be financial or non-financial, professional, or personal. Competing interests can arise in relationship to an organization or another person. Please follow this link to our website for more details on competing interests: [http://journals.plos.org/plosone/s/competing-interests](about:blank)

**Our response**: Thank you for editor’s comment. According to editor’s comment, we revised the manuscript and cover letter.

**Competing interests**

BMI Korea supported BMI 1008 materials and research grants for this research. BMI Korea was only related to providing the products in development, but not related to employment, conultancy, patents and marketed products.

This research was supported by the Basic Science Research Program through the
National Research Foundation of Korea (NRF) funded by the Ministry of Education,
Science and Technology [Grant No. NRF-2020R1C1C1011263].

BMI Korea and NRF had no role in the study design, data collection and analysis, decision to publish, or preparation of the manuscript. This did not alter our adherence to PLOS ONE policies on sharing data and materials.

**(Competing interests section; from line 485 to line 494 on page 25)**

4. Please include captions for your Supporting Information files at the end of your manuscript, and update any in-text citations to match accordingly. Please see our Supporting Information guidelines for more information: [http://journals.plos.org/plosone/s/supporting-information](about:blank).

**Our response**: Thank you for editor’s comment. According to editor’s comment, we added the captions for supporting information file.

1. **Reviewer #1**

**Abstract
Background
-Explain what is BMI1008 in the background of the abstract?
Our response**: Thank you for reviewer’s comment. According to reviewer’s comment, we explain what is BMI 1008 in the **Background** of the **Abstrac**t.

The purpose of this study was to evaluate the analgesic effect of BMI1008 (a new drug containing lidocaine, methylene blue, dexamethasone and vitamin B complex)

**(Abstract section; from line 35 to line 36 on page 3)**

**Introduction
-Dexamethasone is a glucocorticoid that has been used to reduce inflammation and tissue damage in a variety of conditions, including inflammatory bowel disease, rheumatoid arthritis, and some malignancies, and its analgesic and anti-inflammatory effects after surgery have been explored.**

**– Why did you mention inflammatory bowel disease? Is it only inflammatory state for the use of glucocorticoids? The point of this sentence should be the mechanism of analgesic action and effect of glucocorticoids?**

**Our response**: Thank you for reviewer’s comment. We agree with reviewer’s opinion. According to reviewer’s comment, we delete the description for inflammatory bowel disease, rheumatoid arthritis and some malignancies.

**(Introduction section; from line 85 to line 87 on page 5)**

**-Vitamin B complex and 5 NaHCO3 have also been extensively studied for their pain-modulating effects. – How?
Where?//**

**Our response**: Thank you for reviewer’s comment. We agree with reviewer’s opinion. According to reviewer’s comment, we added the reference of studying pain-modulating effects of vitamin B complex and NaHCO3, and we changed these sentence to be more euphemistic.

- Vitamin B complex and pH modified by NaHCO_3_ also showed their pain-modulating effects.

**(Introduction section; from line 87 to line 88 on page 6)**

**Methodology**

**Motor function tests**

**-There were only three rats per group in motor function examination. Why? In all other experiments there were 10-12 rats what is good for statistical significance.**

**Our response**: Thank you for reviewer’s comment. Motor dysfunction due to systemic neurotoxicity following local infiltration was not considered as the primary endpoint of this study when planning this research. The primary endpoint of this study is to measure the analgesic effect of BMI1008. We calculated sample size for the primary endpoint,but did not for secondary endpoint. For this reason, the sample size of experimental rats was not calculated in the motor function test.

**We also drew the response surface analysis plot of the drug combination to summate the analgesic effect of lidocaine and BMI-L. The analgesic effect of each combination was calculated using means of the area under the curve (AUC) for 3 d in Ln(MWT). Moreover, seven points of drug combination were selected when BMI-L was less than 2x and the concentration of lidocaine was less than 2.5%. –**

**-Reference?**

**-Why did you choose these method of examination of the summary of the drugs?**

**Our response**: Thank you for reviewer’s comment. Although the methods for investigating drug interaction (additivity, synergism, and antagonism) are well established in systemic administration, there were few studies investigating drug interaction in local administration. Further, we did not measure the drug concentration in blood or tissues. Therefore, instead of isobologram or NONMEM, we used the response surface model for the purpose of exploring the analgesic effect according to the drug concentration and their combinations.

According to reviewer’s comment, we included a reference in the manuscript.

- We also drew the response surface analysis plot of the drug combination to summate the analgesic effect of lidocaine and BMI-L(14). The analgesic effect of each combination was calculated using means of the area under the curve (AUC) for 3 d in Ln(MWT). Moreover, seven points of drug combination were selected when BMI-L was less than 2x and the concentration of lidocaine was less than 2.5%.

**(Materials and Methods section; from line 285 to line 289 on page 15)**

**Results**

**-It will be more appropriate to show the results of motor function test through graph.**

**Our response**: Thank you for reviewer’s comment. According to reviewer’s comment, we added a graph **(Figure 7)** in the manuscript**.**

**Response surface analysis**

**The AUC of Ln(MWT) showed an approximately linear positive correlation with BMI-L and lidocaine concentration. Moreover, co-administration of BMI-L and lidocaine augmented the AUC of Ln(MWT) compared with the administration of single drugs. Gross concavity of the plane was not observed, suggesting a higher possibility of the additivity assumption and a lower possibility of the antagonism assumption (Figure 6).**

**- How did you conclude this? Is it based on some previous work? Why did you choose this method? There are a lot of others more concrete methods for the interactions of the drugs. Maybe, it would be better to add some other method for this part of results.**

**Our response**: Thank you for reviewer’s comment. As we mentioned above, we used this methods because there was few previous works in local infiltration and we did not measure drug concentration in blood or tissues. To add a conceptual explanation, a figure related to the response surface model is attached here.

In our figure, we cannot observe gross concavity of the plane. Therefore we thought it suggests possibility of the additivity assumption more likely, and the antagonism assumption lesser likely.

**Discussion**

**The additivity of lidocaine and BMI-L appeared more likely than antagonism when these drugs were co-administered.**

**-The same remark as I mentioned in the results.**

**Our response**: Thank you for taking your precious time to review the manuscript. According to your advice, we added a review article on the methodology as a reference.

- We also drew the response surface analysis plot of the drug combination to summate the analgesic effect of lidocaine and BMI-L(14). The analgesic effect of each combination was calculated using means of the area under the curve (AUC) for 3 d in Ln(MWT). Moreover, seven points of drug combination were selected when BMI-L was less than 2x and the concentration of lidocaine was less than 2.5%.

**(Materials and Methods section; from line 285 to line 289 on page 15)**

Thank you again for you to take your precious time to review our work.

2. **Reviewer #2**

**Paper titled (Effects of a BMI1008 Mixture on Postoperative Pain in a Rat Model of Incisional Pain)**

**- Generally, the authors did very hard work, I read it more time but it is confusing so if they try to simplify the measurements and the result**

**Our response**: Thank you for your comment for the betterment of our manuscript. According to your comment, we tried to simplify the measurement and the result **(Results section)**

**- Is the BMI1008 combination is approved or registered**

**Our response**: Thank you for reviewer’s comment. BMI1008 is still in the development stage for proper drug combination and was not approved or registered. The experimental agents including BMI-L and BMI1008 concentrates were provided from the manufacturer. The manuscript has been revised to clarify this point.

**🡪** a newly developed experimental drug

- The manufacturer provided experimental agents including BMI-L and BMI1008 concentrates.

**(Introduction section; from line 89 on page 5, Materials and Methods section; from line 111 to line 112 on page 7, Competing interests section; from line 485 to line 494 on page 25)**

**- the combination of more than one drugs may result in neurotoxicity**

**Our response**: Thank you for reviewer’s comment. We agree with reviewer’s comment. According to reviewer’s comment, we revised the manuscript.

- No case for neurotoxicity was found in our study. However, possibilities for neurotoxicity from combining more than one drug cannot be ruled out entirely.

**(Discussion section; from line 461 to 463 on page 24)**

**- Is vitamin b has local effect on nerve cells or axone?**

**Our response**: Thank you for reviewer’s comment. As we described in the manuscript, there have been many studies investigating nerve conduction, excitability and pain disorders. (**Discussion section; from line 451 on page 23 to line 459 on page 24)** However, almost studies investigating the effect of systemic administration of vitamin B. Therefore the effect of local administration of vitamin B was not known. We agree with reviewer’s opinon, thus we have a plan a histologic study to investigate the effect of local administration of vitamin B.

Thank you again for reviewer’s opinion.

3. **Reviewer #3**

**This is an experimental study to confirm the control of incisional pain according to the dose of BMI1008 and BMI-L. However, authors need to correct the manuscript overall.**

**Our response**: Thank you for your comment for the betterment of our manuscript.

**MP4, L84-85 . Please add a reference.**

**Our response**: Thank you for reviewer’s comment. According to reviewer’s comment, we add the references of studying pain-modulating effects of vitamin B complex and NaHCO3.

- Dexamethasone is a glucocorticoid that has been used to reduce inflammation and tissue damage in a variety of conditions, and its analgesic and anti-inflammatory effects after surgery have been explored (6). Vitamin B complex and pH modified by NaHCO_3_ also showed their pain-modulating effects (7, 8)..

**(Introduction section; from line 85 to line 88 on page 6)**

**MP18, L390 "The additivity of lidocaine and BMI-L appeared more likely than**

**antagonism when these drugs were co-administered." Can this dose-response relationship be explained as antagonism? I cannot understand. Please explain more detail.**

**Our response**: Thank you for reviewer’s comment for the betterment of manuscript. This dose-response relationship was explained as ‘additivity’ rather than ‘infra-additivity(antagonism)’. I agree to reviewer’s comment that the reader may be confused about the terminology of drug interaction. Thus, we edited the sentence to make the author's intentions clearer.

The additivity of lidocaine and BMI-L appeared more likely than antagonism when these drugs were co-administered. (Discussion section, page 18, line 390)

- Drug interaction between lidocaine and BMI-L were more likely to be additivity than antagonism (infra-additivity) when these drugs were co-administered.

**(Discussion section; from line 395 to line 396 on page 21)**

**The discussion section is not organized in general. Rearrange all paragraphs. For example, you need not to elaborate on the (MP18, L396-399) incisional pain model. Rather, it would be better to find the literature on the dose-response relationship of drugs and add them. Or please add more details such as analgesic effect when used in actual anesthesia clinical practice**

**Our response**: Thank you for reviewer’s comment for the betterment of manuscript. According to your advice, we revised the discussion section. We deleted the paragraph for incisional pain model, and added the literature on the dose-response relationship. We also rearranged the paragraphs to have more logical order. Thank you again for your precious comment.

**MP19 L410 It has been reported that vasoactive agents and alpha2-adrenergic agents increase the quality and duration of local anesthetics. For example, which drugs do you mean? Are there any drugs other than epinephrine that have such an effect?**

**Our response**: Thank you for taking your precious time to review the manuscript. The alpha2-adrenergic agents such as clonidine and dexmedetomidine are clinically used to enhance the analgesic effect. According to your advice, we added reference to the discussion section

Thus, adjuvant drugs, such as opioids, vasoactive agents, alpha2-adrenergic agonists, steroids, and nonsteroidal anti-inflammatory drugs, have been applied to decrease the toxicity of the local anesthetics while increasing the quality and duration of anesthesia and analgesia (18, 19). **(Discussion section, page 21, line 407 to line 410)**

**MP21 464 Is it correct to use the word "hyperalgesia"? Do you mean "mechanical stimulation"?**

**It is very important to write in the correct terminology.**

**Our response**: Thank you for taking your precious time to review the manuscript. In the field of pain research, hyperalgesia and allodynia are widely used terms.

(DOI: https://doi.org/10.1016/S1474-4422(14)70102-4

URL: https://www.thelancet.com/journals/laneur/article/PIIS1474-4422(14)70102-4/fulltext)

In this study, mechanical hyperalgesia was measured as the mechanical withdrawal threshold.

Thank you again for reviewer’s comment for the betterment of manuscript.

**I have a question, are BMI1008 and BMI-L drugs that the authors prepared and used in the study? Is it a drug marketed under a brand name?**

**Our response**: Thank you for reviewer’s comment. BMI1008 is still in the development stage for proper drug combination and has yet to be approved or registered. The experimental agents including BMI-L and BMI1008 concentrates were provided from the manufacturer. The manuscript has been revised to clarify this point.

The manufacturer provided experimental agents including BMI-L and BMI1008 concentrates. **(Introduction section; from line 89 on page 5, Materials and Methods section; from line 111 to line 112 on page 6, Competing interests section; from line 485 to line 494 on page 25)**

We hope this revised manuscript will better meet the requirement of the *‘Plos one’* for publication. We heartily thank you for your constructive review.

Sincerely yours,

Hyun Kang, M. D., Ph. D., M.P.H.

Professor

Department of Anesthesiology and Pain Medicine

Chung-Ang University College of Medicine

84 Heukseok-ro, Dongjak-gu, Seoul, 06911, Korea

Tel: +82-2-6299-2571, 2579, 2586 / Fax: +82-2-6299-2585

E-mail: [roman00@naver.com](mailto:roman00@naver.com)
